# Dissecting the role of EYS in retinal degeneration: clinical and molecular aspects and its implications for future therapy

**DOI:** 10.1186/s13023-021-01843-z

**Published:** 2021-05-17

**Authors:** Ana B. Garcia-Delgado, Lourdes Valdes-Sanchez, Maria Jose Morillo-Sanchez, Beatriz Ponte-Zuñiga, Francisco J. Diaz-Corrales, Berta de la Cerda

**Affiliations:** 1 Andalusian Center for Molecular Biology and Regenerative Medicine (CABIMER), Avda. Americo Vespucio 24, 41092 Seville, Spain; 2 Department of Ophthalmology, University Hospital Virgen Macarena, Seville, Spain; 3 Retics Oftared, Institute of Health Carlos III, Madrid, Spain

**Keywords:** Retinal dystrophy, *EYS*, Ciliopathy, Animal models, Advanced therapies

## Abstract

Mutations in the *EYS* gene are one of the major causes of autosomal recessive retinitis pigmentosa. EYS-retinopathy presents a severe clinical phenotype, and patients currently have no therapeutic options. The progress in personalised medicine and gene and cell therapies hold promise for treating this degenerative disease. However, lack of understanding and incomplete comprehension of disease's mechanism and the role of *EYS* in the healthy retina are critical limitations for the translation of current technical advances into real therapeutic possibilities. This review recapitulates the present knowledge about EYS-retinopathies, their clinical presentations and proposed genotype–phenotype correlations. Molecular details of the gene and the protein, mainly based on animal model data, are analysed. The proposed cellular localisation and roles of this large multi-domain protein are detailed. Future therapeutic approaches for EYS-retinopathies are discussed.

## Background

Retinitis pigmentosa (RP, OMIM #26800) is the most common form of inherited retinal degeneration (IRD), with an estimated prevalence of 1 per 4,000 people. Although RP is a rare disease, it represents the primary cause of hereditary blindness in adults, affecting more than one million people worldwide [1]. *EYS* is a major causative gene for autosomal recessive RP (arRP) [2] in all ethnicities. EYS-retinopathy manifests early in life and produces a severe disability, currently without therapeutic options. The study of the disease's molecular mechanism has been hampered by the lack of a representative animal model for this human IRD. Information on the cellular localisation and molecular features of EYS, obtained from different vertebrate animal models, is summarised in this review to get insight into this protein's possible roles in the human retina.

Gene therapy is emerging as a safe and effective treatment for some types of RP caused by specific genes such as *RPE65*. Future therapeutic approaches for EYS-retinopathies are discussed based on this large gene's limitations and the current advanced therapies state-of-the-art.

## Main text

**Fig. 1:**
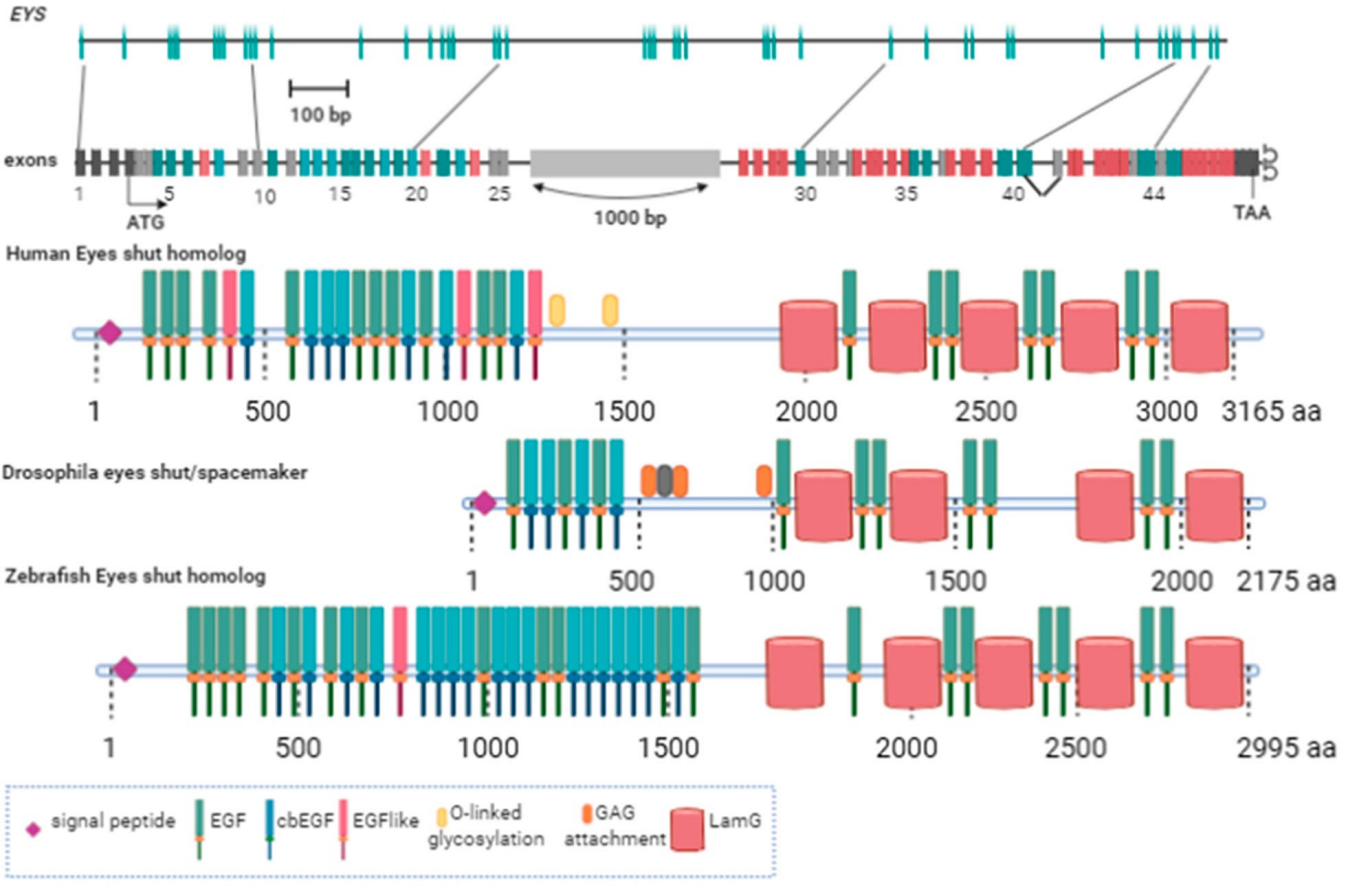
Human EYS genetic structure and domain composition of the protein predicted from the full-length transcript compared to Drosophila and zebrafish orthologues**.** Some conserved posttranslational modification sites are marked

The Eyes shut homolog (*EYS*) gene was identified as the causing gene for retinitis pigmentosa 25 (RP25) (OMIM 612424, NM_001142800) in 2008 [2]. The gene is noted as the largest one expressed in the human eye, extending for 2 Mb of genomic DNA in chromosome 6 (6q12.1-6q15) [2]. The genetic structure initially described for *EYS* included 43 exons, coding for a multi-domain protein of 3,145 amino acids with 21 N-terminal epithelial growth factor (EGF)-like and 5 C-terminal Laminin (Lam) G domains, interspaced with additional EGF-like domains [2] (Fig. 1). The domain organisation of *EYS* makes it an orthologue of the Eys gene of Drosophila melanogaster, which encodes the Spam protein, expressed in the open rhabdomere eye of a range of insect species [3, 4].

An independent study of the RP25 locus concurrently identified the *EYS* gene as causative for RP25 [5], describing a slightly different gene structure, with an additional 63 bp exon after exon 41, making a total of 3,165 amino acids for the full-length protein. The extra exon is the subject of alternative splicing. A large *EYS* transcript was identified, with 10,475 nucleotides, including the untranslated 3′ and 5′ regions. Both initial reports found that the new gene was preferentially and abundantly expressed in the human retina, although minor expression was also found in other tissues [2, 5].

Unfortunately for the study of the associated disease, *EYS* is missing or interrupted in the genome of several lineages, including the most common animal models: mouse, rat and guinea pig [2]. Regarding the protein function, a direct correlation with its ortholog in Drosophila cannot be assumed since rhabdomeres and photoreceptors have very different cellular structures. Additionally, the lack of sequence conservation in some key residues points to functional differences. Some causative mutations localise in the ten C-terminal residues in patients, not conserved with Drosophila but well conserved among vertebrates [5]. Instead, the fly's gene has a series of conserved sites for glycosylation absent in the human gene. Zebrafish have been used as an alternative vertebrate model to study the role of Eys protein. The fish gene shares a high degree of similarity with the human gene in its structure (Fig. 1) and sequence. Although the predicted protein is short, the missing part corresponds to a low-complexity region that is also missing in other vertebrates with a functional Eys protein [6].

### Clinical manifestations

After identifying the *EYS* gene, patients presenting retinal dystrophy without a genetic diagnostic were tested for mutations in this gene, revealing it as the major gene for non-syndromic arRP. *EYS* has been found in all ethnicities studied, with a prevalence ranging from 5 to 33%, depending on the population. It represents 5% of arRP in the Dutch and Canadian population, from 5 to 16% in Europe and 18–23.5% in the Japanese cohorts, where *EYS* is the most frequent cause of IRD [7–16].

RP25 is a severe form of arRP with equal prevalence among males and females. Visual acuity loss usually starts in the second or third decade of life, with a frequent early manifestation as night blindness and a progressive visual field constriction. Patients usually preserve central vision until very late in life [14, 17]. In some cases, the initial presentation includes photophobia instead of night blindness [18]. Relatively young RP25 patients often present subcapsular cataracts and benefit from cataract surgery, even with minor opacities [14, 18].

**Fig. 2:**
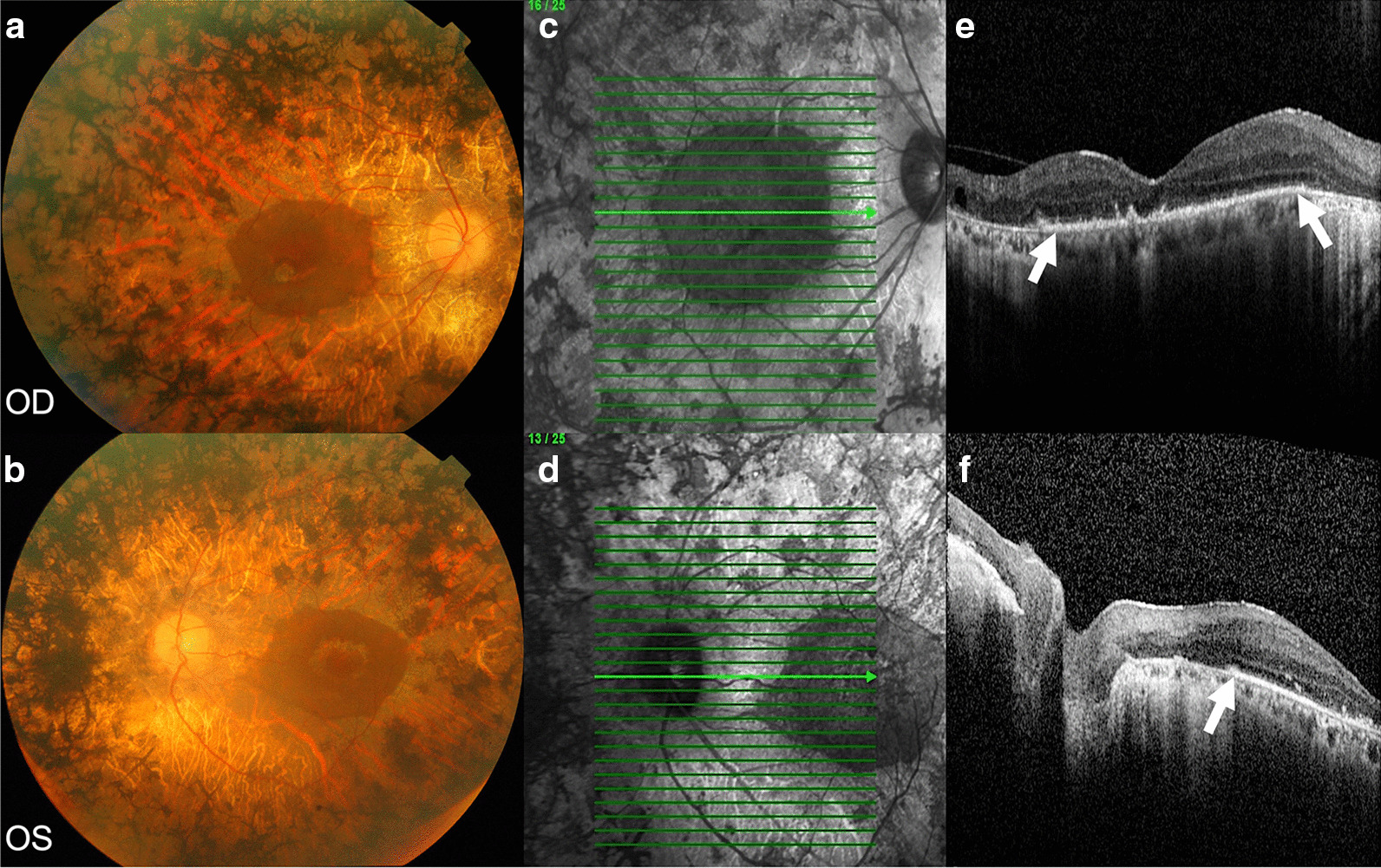
Fundus photography (**a**, **b**), infrared (IR) (**c**, **d**) and optical coherence tomography (OCT) (E, F) of a male patient (age 78) diagnosed with retinitis pigmentosa (RP). Homozygous *EYS* variant c.5928-2A>G was detected in this patient using whole-exome sequencing. The electroretinogram displayed cone and rods extinguished responses. Best-corrected visual acuity (BCVA) OD + 1 logMAR and OS hand motion at 1 m. Biomicroscopy of anterior pole: OU pseudophakia. Fundus photography shows a typical RP phenotype (**a**, **b**). The optic disk appears pale, vessels are very thin, and there is extensive atrophy of the retinal pigment epithelium and choriocapillaris with bone spicule pigmentations. Preservation of central retinal islet with a perifoveal orange halo. **a**, **b** The IR image shows the retinal islet's detail and the scan lines where the OCT was performed (**c**, **d**). OCT scans display the bilateral perifoveal retinal islet. The ellipsoid zone is preserved and limits the retinal islet (**e**, **f**; arrows)

Regarding ophthalmic data, RP25 usually fulfils all the classic RP hallmarks, with fundus images of bone spicule pigmentation, optic disc pallor, retinal vessel attenuation (Fig. 2) and reduced or non-detectable electroretinogram (ERG) response under both scotopic and photopic conditions. In the initial phases of the disease, central retinal layers appear preserved in the optical coherence tomography (OCT). The atrophy observed is restricted to the retinal pigment epithelium (RPE) and the mid and peripheral retina choriocapillaris. As the disease progresses, macular region thinning is apparent. Fundus autofluorescence (FAF) imaging has demonstrated to help determine the disease's extent and facilitate diagnosis in atypical cases. In early RP25, the peripheral ellipsoid zone (EZ) is less visible, and the edge of EZ can approximate the visual field along with disease progression [17].

The disease's clinical features correlate with the histological findings in a study of post-mortem eyes [19]. In this report, donors were of advanced age; therefore, observations correspond to advanced RP25: Gliosis and disorganisation of the retinal structure, particularly in the periphery, with very few photoreceptors present, along with some isolated areas of preserved cones and RPE in the perifovea, possibly explaining the remaining central vision, as shown in Fig. 2. Opsin pigments were mislocalised in the surviving cone cells, found mainly in the inner segments and cell bodies instead of outer segments (OS) [19]. Cell migration to inner retinal layers, indicative of a degenerative tissue remodelling process, has also been observed in OCT examination of advanced EYS-retinopathy [14].

Initially, *EYS* mutations were only associated with RP [5, 14], being the most common type of clinical presentation (90%). However, macular dystrophy and autosomal recessive cone-rod dystrophy (arCRD) are other possible manifestations of EYS-retinopathy [16, 20–22]. EYS-arCRD has been reported with a low frequency (1%) [22], manifesting with a loss in central vision, predominant degeneration of the macular region and decreased amplitudes in the cone and rod ERG waves [20]. EYS-arCRD has been described in a patient with the same compound heterozygous *EYS* mutations [22] that cause arRP in other cases, implying the existence of unknown factors that modify the clinical phenotype. There is not enough genetic data to determine the prevalence of *EYS* mutations among CRD patients [23]. However, it is relevant to note that the retinal degeneration pattern in eys knockout zebrafish, the most used vertebrate model for EYS-retinopathy, is similar to CRD [24].

### Genotype–phenotype correlation

The array of disease-associated mutations reported for *EYS* includes insertion, deletion, nonsense or missense substitution and splice site mutation, with variable representation in different cohorts [2, 5, 8, 9, 14, 18, 25, 26]. The most common mutations in *EYS* are truncating mutations that lead to premature stop codons, removing LamG or EGF-domains [13], including frameshift and nonsense mutations. Missense mutations have also been described and are usually found in combination with a truncating mutation [8, 9, 14, 18, 25, 27]. Additionally, midsized genomic rearrangements cause 4% of EYS-arRP and participate as the second pathogenic variation in 15% of cases [28]. Compound heterozygous inheritance of two different copy-number variation (CNV), or a CNV and a nonsense mutation in *EYS*, have also been described [9, 29, 30].

Several authors have tried to link the type of mutation with the clinical presentation, but different trends have been found depending on the cohort studied. Iwanami and cols. [13] associated truncating mutations to a severe decline in visual acuity. The explanation would include the nonsense-mediated decay (NMD) of the truncated transcripts, leading to decreased EYS protein dose. McGuigan and cols. [26] also proposed NMD, based on the relatively uniform location of truncating mutations along the gene sequence and the observation of milder cases associated with truncating mutations that affect the last C-terminal EGF and LamG domains. These authors proposed that the mutations would result in hypomorphic alleles, in which mRNA may avoid NMD, producing a truncated EYS protein with some residual functionality. In another cohort, studied by Bonhilla and cols. [19], midsize exonic deletion correlated with better preservation of macular cones and central vision than truncating mutations. Regarding missense mutations, they can be found as compound heterozygote with another missense mutation or a truncating mutation. It is currently under debate if missense changes produce a less severe disease phenotype than truncating mutations. Contradictory data from different cohorts keep this question open. Moreover, the effect of missense mutations could be influenced by additional single nucleotide polymorphisms (SNPs) in the *EYS* gene or interaction with other modifier factors in the genetic background that might affect the phenotype. The pathogenicity of many missense changes remains unclear, even for conserved residues [31].

Regarding the gene sections affected by the mutation, it has been reported a higher frequency of disease-associated mutations close to the C-terminus [5, 9, 11]. These observations and the fact that some C-terminal domains are highly conserved support the EYS C-terminal region's importance for protein functionality. On the contrary, Ogino and cols. [17] suggested that variants near the distal portion of the protein will have a minor effect on the protein function, only impairing EYS binding with its interactome. In a report covering more than 200 *EYS* mutations [26], a homogeneous distribution of mutations along the gene sequence was described, with a uniform spread of splice site and truncating mutations. Differences were found in missense mutations, more abundant in the C-terminal region, and in deletions, mainly found in the first half of the protein. The significance of this data is still unclear since many deletions also result in premature stop codons. The type of mutation cannot be easily related to the phenotype's severity, as some patients with a relatively mild disease harbour truncating mutations. In contrast, others with missense mutations present a severe disease [32]. In the study of Sengillo and cols. [16] patients were classified regarding fundus FAF: a crescent-shaped autofluorescent ring was associated with C-terminal mutations whilst the typical autofluorescent ring in the distal area, characteristic of RP patients, was found in patients with N-terminal mutations.

Moreover, phenotypic variability among patients with the same mutations has also been shown [5, 32], indicating the input of other contributing factors to the clinical presentation and preventing the use of the genetic diagnostic to predict the disease's evolution and severity. Moreover, all EYS patients preserve a normal visual acuity until their second decade of life means that EYS protein is not essential in retinal development. Some unknown factor may compensate for the EYS defect, allowing for visual function in the first decades of life. It has been already mentioned that the *EYS* gene is lost in several species, in which its function has to be played or compensated by other factors. The possible role of additional genetic modifiers adds a layer of complexity to the understanding of genetic implications on the disease's prognosis.

Another significant gap in current knowledge that impairs the prediction of the mutations' effect is the incomplete understanding of EYS function in the healthy retina, preventing the prediction of the possible effect of mutations in conserved residues or those affecting functional domains. To better understand EYS-retinopathies' pathophysiology, it is essential to perform functional studies that focus on the effect of mutations on the different EYS isoforms and their impact on the retina. In a cellular modelling study [33], photoreceptor–directed fibroblasts from EYS-RP patients were used to evaluate the effect of different mutations on EYS transcripts, finding that level of NMD depends on the specific mutation.

In summary, the variability in the severity of missense mutations and the lack of hotspots in *EYS* make genotype–phenotype relationships challenging to predict. Being *EYS*, a gene of high prevalence in IRD, the analysis of larger cohorts comprising a wide array of mutation types and longitudinal studies on the natural course of the disease would add vital information to predict the clinical evolution and prognosis of genetically diagnosed patients.

### Molecular aspects

Genomic and protein databases predictions based on *EYS* gene structure [2, 5] depict a full-length protein structure divided into two distinct halves: a N-terminal part having a signal peptide followed by a stretch of EGF-like domains and a C-terminal part rich in LamG domains interspaced with additional EGF-like domains (Fig. 1). Proteome dynamic regulation via splicing is conserved in vertebrate neurons and contributes to the nervous system's complexity [34]. Concerning EYS, a growing number of variant transcripts have been identified, resulting from alternative splicing [35, 36]. Expression of different isoforms has been detected in the retina and other tissues. The diverse EYS isoforms, with particular domain composition and structure, will perform specific roles depending on time, cellular compartment or tissue, complicating the analysis of the effect of mutations. Isoforms expressed in the human retina and their main characteristics can be found in Table 1.

**Table 1:**
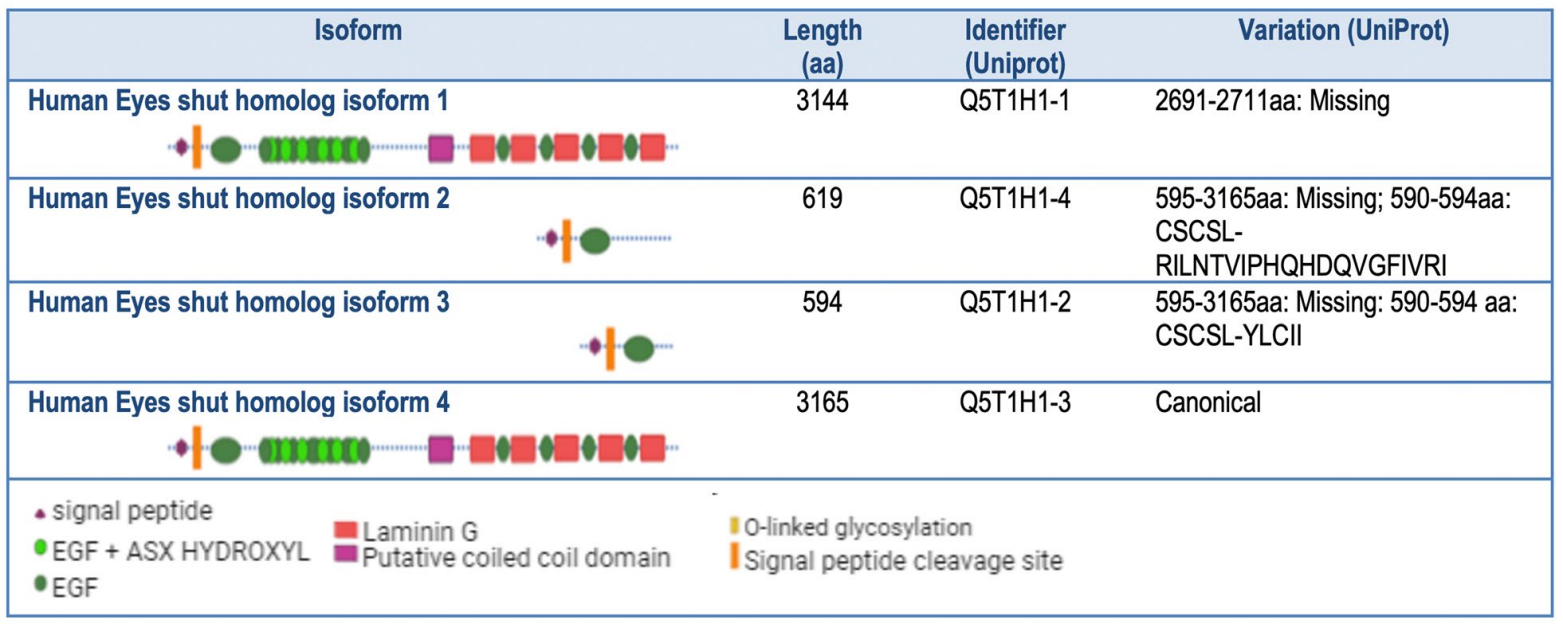
EYS isoforms expressed in the human retina.

The longer isoforms 1 and 4 only differ in the insertion of one exon between exons 41 and 42. High expression of these longer isoforms was found in the retina in the initial descriptions of the *EYS* gene [2, 5]. Isoforms 2 and 3 correspond to the 5′ part of the gene, comprising the signal peptide and five EGF-like domains, with some differences in their C-terminal section's length and sequence. Isoforms 2 and 3 are expressed in the retina and testis [35].

All the retinal transcripts known for EYS enclose the signal peptide and longer or shorter stretches of the 5′sequence of the gene. Transcripts containing the 5′ segment of the gene are conserved among distant vertebrate species [35, 36]. A fifth transcript, including only 3′ sequences, starting from exon 37 and containing two LamG domains and EGF-like domains, was recently identified in human dermal fibroblasts [36]. This transcript without signal peptide presents some variation due to splicing, generating isoforms 5–1, 5–2 and 5–3. These isoforms are predicted to be expressed ubiquitously, mainly in hepatocyte, bile duct, prostate, myoblast, lung, kidney and intestinal epithelial cells [36].

The two distinct halves of the full-length EYS protein will likely have different roles and interactors. Shorter isoforms with just the EGF-rich or the LamG-rich sections will probably have particular localisations and functions, which will be relevant for analysing and predicting the phenotypic impact of genetic variants.

### Functions of EYS and possible mechanisms of the disease

**Fig. 3:**
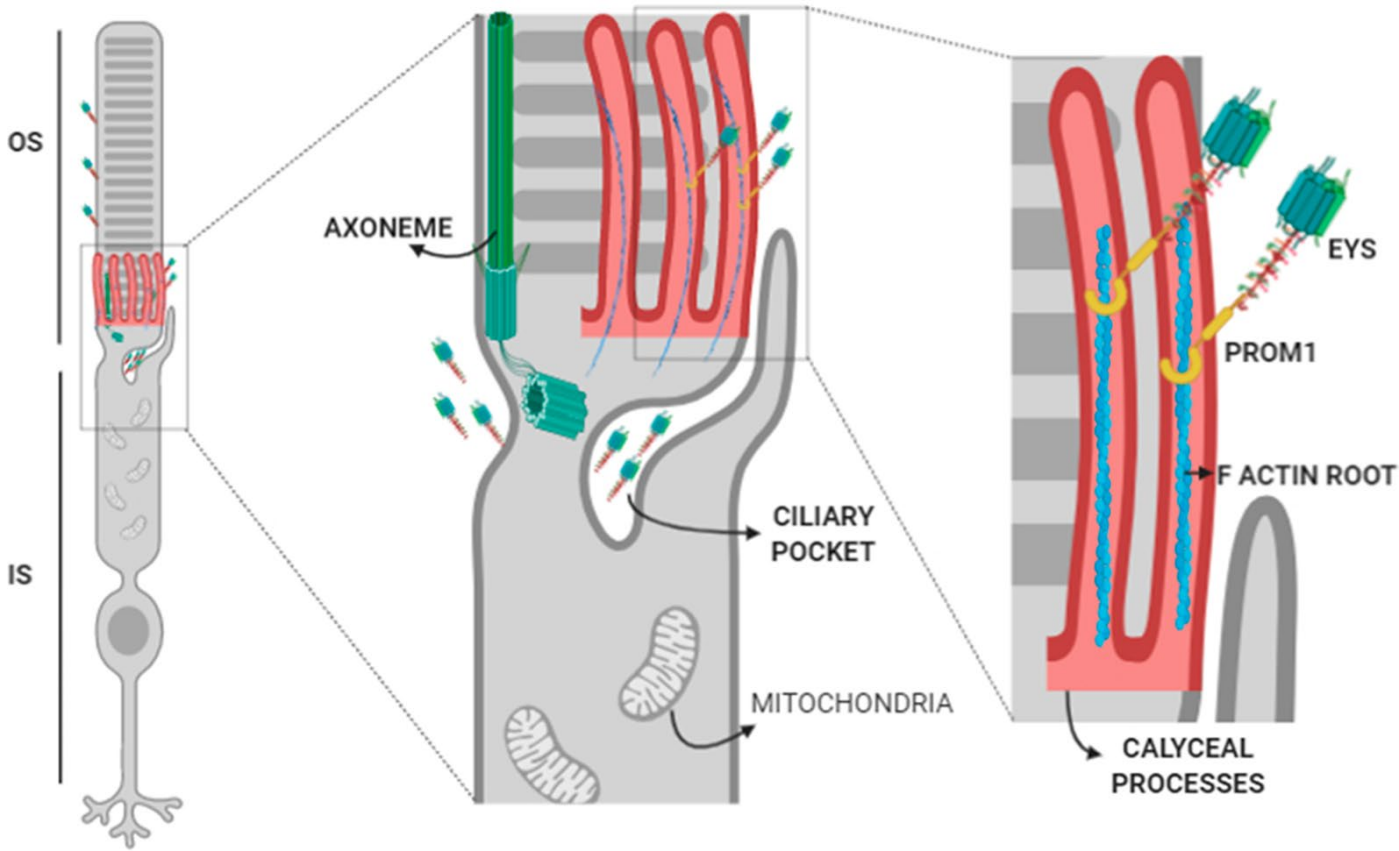
Possible localisation and interactions of EYS in the transition zone of photoreceptors. The extracellular protein is found in the IPM close to the primary cilium and the OS. A proposed localisation in the ciliary pocket and an interaction with Prom1 in the calyceal processes are shown

**Fig. 4:**
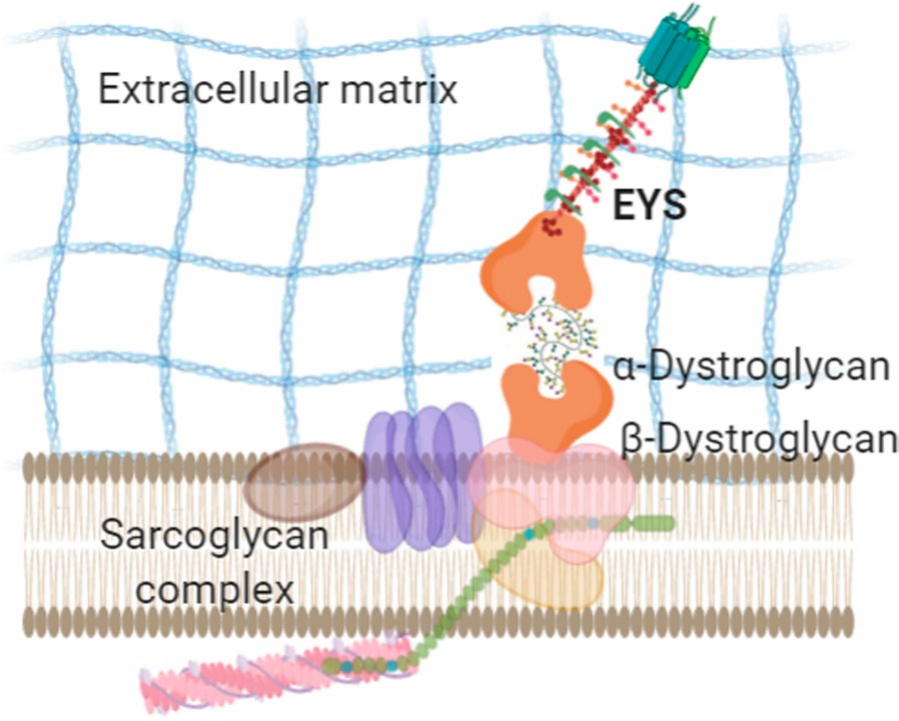
Graphic representation of the proposed interaction between EYS and the ECM components

**Fig. 5:**
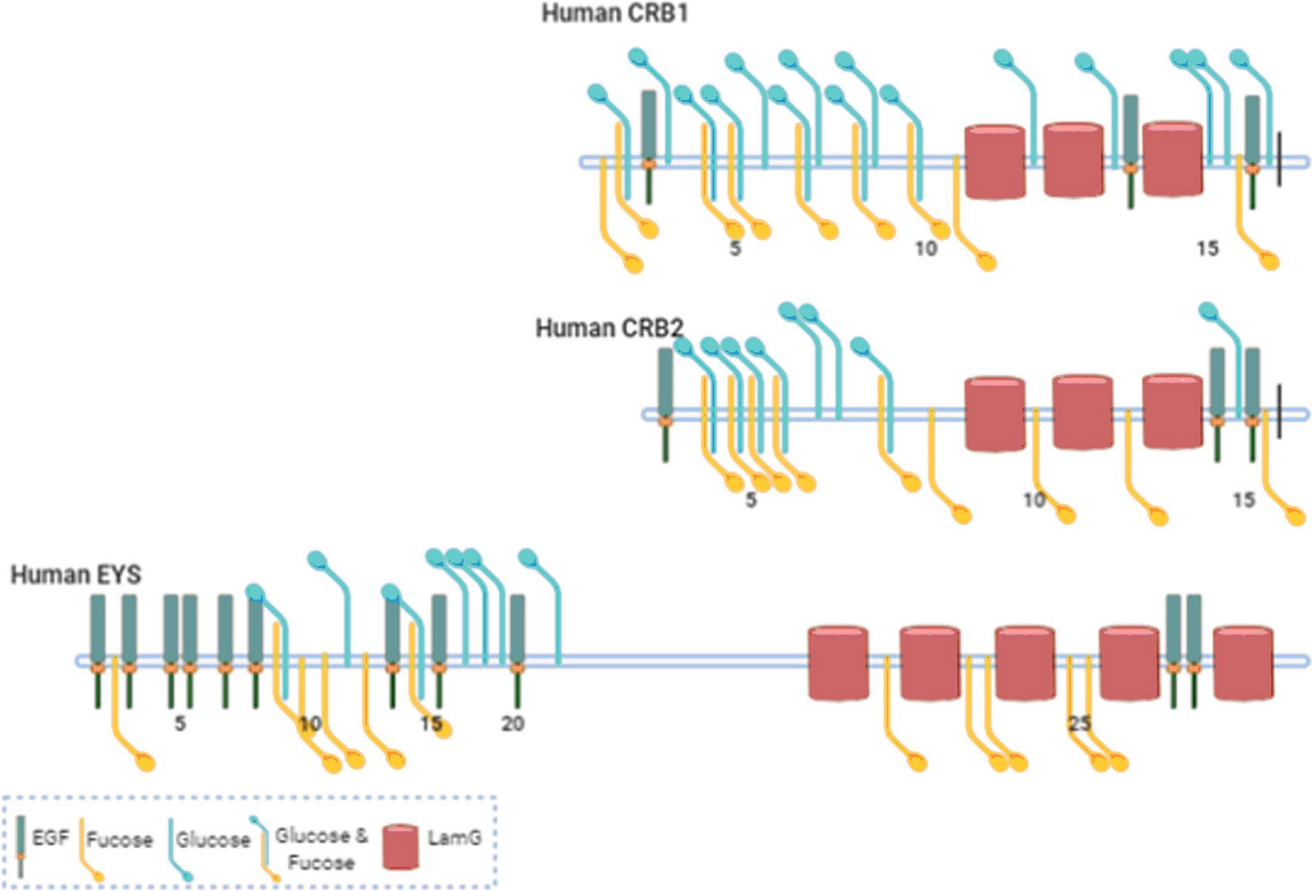
O-glycosylation sites in human EYS, CRB1 and CRB2 proteins

- **EYS localisation and ciliary role**
  Most of the knowledge about this gene comes from its ortholog in Drosophila (*eys* gene; eyes shut protein), an extracellular protein essential to maintaining the inter-rhabdomeric space [37]. It has also been localised to the extracellular space of the fly's mechanosensory neurons, supporting the sensory cell [3]. Although the two orthologs share structural similarities, some molecular differences will affect posttranslational modifications such as glycosylation, impacting protein localisation and function. Together with the critical structural differences between the fly and vertebrate eyes, these facts limit the utility of the information on localisation, interactions, and function of eys to understand human EYS role.
  Prominin is a highly glycosylated transmembrane protein known to localise in membrane protrusions of many cells. In the retina, it is found in the membranous protrusions of the OS base, which in zebrafish and human are organised as a ring of calyceal processes. Mutations in *PROM1* cause arRP and macular degeneration [38–40], and its function has been described as essential for membrane structure and disc morphogenesis. Prominin interacts with actin filaments and protocadherin21, also localised to the OS bottom [40].
  It has been shown [41] that the interaction between Prominin and Eys is genetically conserved between Drosophila and human. Human *PROM* and *EYS* were able to replace the fly's mutant proteins, rescuing the morphology of the rhabdomere [41], suggesting that the interaction between these two partners has been evolutionarily conserved and, thus, may still be playing a role in vertebrates.
  The localisation of the full-length Eys protein was first reported in the OS of photoreceptors in pig retinal Sects. [25] and further refined to the proximity of the ciliary axoneme of rod and cones. Another study reported Eys' localisation in the cytoplasm of ganglion cells in macaque retinas [35]. Further details were provided in a concurrent study [42] in which some differences in cellular localisation of Eys were found between different vertebrate retinas. In zebrafish, Eys was found in the connecting cilium/transition zone area. In macaque retinas, most of Eys labelling was found in the interphotoreceptor matrix (IPM) surrounding cone OS and stained with less intensity the OS of rods and the region between OS and RPE. In both photoreceptor types, Eys localised in the proximity of the connecting cilium/transition zone. Although the most frequent form of EYS-retinopathy is rod dystrophy, it has been shown that cones also express this protein, which might account for EYS-arCRD cases.
  Even with the interspecific variability described, the value of zebrafish as a model for the study of EYS is apparent. Knocking eys induces retinal degeneration in the fish [6, 42]. A detailed description of the morphology of mutant photoreceptors by electron microscopy showed the collapse of the ciliary pocket, formed by a plasma membrane invagination that separates the outer and inner segments. As expected from its sequence, Eys was localised as an extracellular protein in the IPM. Functionally, Eys demonstrated to be essential for the structural maintenance of the ciliary pocket. Another study in zebrafish reported visual impairment when eys was mutated, along with mislocalisation of proteins of the OS of the photoreceptors and distortion of F-actin filaments, mainly affecting cones [24]. Considering that actin filaments are essential for protein transport, this might explain the mislocalisation of OS proteins. It is to note that eys zebrafish mutants present a disruption in their calyceal processes. These cellular structures are actin-filled microvilli anchored to the inner segment by F-actin roots and superficially encircling the OS base. Although their function has not been determined, it has been proposed that these microvilli provide mechanical strength to the base of the OS, a part of the photoreceptor cell subjected to physical stress due to the continuous synthesis of new discs. The calyceal processes have been described in photoreceptors of zebrafish, frogs, pig and primates, including human, but are absent in mouse retinas [43]. The presence of calyceal processes curiously coincides with eys gene distribution among these vertebrate species. In a parallel case, USH1 proteins, including Cadherin-23, localise to the calyceal processes, which in macaques and Xenopus present F-actin roots. Mouse and rat animal models, devoid of calyceal processes and F-actin roots in the OS base, do not phenocopy the retinal features of *USH1* mutations.
  The absence of calyceal microvilli structures in mouse and rat and the fact that they become disorganised in the standard tissue fixing conditions has undermined these structures' study. However, it might be worth focusing on the interactions between F-actin roots of calyceal processes, its associated proteins such as Protocadherin23, the transmembrane Prominin and the large protein associated with the IPM Eys in the maintenance of the membranous structures of the transition zone of vertebrate photoreceptors. Considering what is known for Drosophila, where mutations in the *eys* gene collapse the matrix-filled inter-rhabdomeric space, the finding of Eys defects as causative for the vertebrate's shrink ciliary pocket suggests further functional homologies between the fly and the vertebrate proteins. These similarities might be extended to EYS interaction with PROM1. A hypothetic network of proteins comprising EYS, PROM1 and ACTIN can be proposed to be involved in the structural maintenance of the photoreceptors' transition zone (Fig. 3). Prominin is known to be involved in the structural organisation of cellular protrusions, interacting with F-actin filaments and cadherins and has been localised to the OS base. Although *PROM1* mutations cause retinopathy, the role of this protein in photoreceptors is poorly studied [44].
  Summing up from the currently available data, the EYS function is essential in the structural organisation of the connecting cilium of vertebrate photoreceptors. Approximately 25% of all IRDs can be categorised as ciliopathies [45], but EYS is the first extracellular protein known to cause ciliopathy. Even so, it cannot be ruled out for some EYS isoforms to have additional extracellular roles in the conformation of the IPM that fills the space between photoreceptors and between them and RPE.
- **LG domains of EYS and the interaction with the IPM**
  The IPM is mainly composed of glycoproteins and proteoglycans and has an essential function in the structural maintenance of tissue, in the transport and recycling of cellular components, and in the maintenance of photoreceptor differentiation state and survival. *EYS* is one of several non-syndromic RP causing genes that encode a protein that binds hyaluronic acid and localises in the IPM. The EYS protein contains LG domains that make it a candidate to bind glycosaminoglycan chains, and some of its possible roles may be mediated through interactions with matriglycans.
  Matriglycan is a linear chain of repeating disaccharides found in dystroglycan (DG) in the cell surface. DG is formed by extracellular α-DG and transmembrane α-DG that interacts with the cytoskeleton. Agrin, laminin, perlecan, neurexin and pikachurin are extracellular α-DG-interacting proteins through their LamG domains [46–49]. Pikachurin, specifically, is essential to maintain the photoreceptor ribbon synapsis [49]. It has been proposed that EYS could interact with matriglycan through its LamG domains [50].
  In zebrafish, it has been demonstrated that EYS binds the O-mannosyl-glycan matriglycan [50]. A reduction in the level of matriglycans through a mutation in Pomgnt1, the O-mannose β1,2 N-acetylglucosaminyltransferase 1, results in EYS mislocalisation from the connecting cilium area to synaptotagmine-1 positive secretory vesicles. The phenotype of these mutant fishes includes retinal degeneration with ageing. In humans, hypomorphic mutations in POMTGnT1 cause RP76 [51, 52]. The clinical manifestation of a dysfunctional POMGTn1, leading to retinal dystrophy, might be mediated by a defective interaction between EYS and the extracellular matrix. A graphic representation of this possible interaction is shown in Fig. 4.
  Although many mouse models with different matriglycan-related mutations phenotypically present retinal defects [49, 53–57], the *Pomgnt1* mutation in mouse does not produce an RP76-like phenotype like in zebrafish, pointing to an effect mediated by *EYS* [50]. It is yet to be established whether another matriglycan-binding protein plays the function of EYS in mouse, or if mouse photoreceptors have some structural adaptation to compensate for Eys' loss.
- **EGF-like domains of EYS and Notch signalling**
  EGF-like domains contain 30 to 40 amino acids with sequence similarity to the EGF protein and, structurally, present three internal disulfide bonds. These domains are usually found as tandem repeats in secreted proteins or the extracellular domains of transmembrane proteins and are involved in protein–protein interactions [58]. The EGF-repeats participate in Notch signalling, a conserved signalling pathway essential for animals' development and health [59]. In mammals, Notch receptors have up to 36 EGF-repeats with diverse functional roles in protein folding, trafficking or interaction between Notch ligands and receptors [60, 61]. Structural similarity of EYS-EGF domains with Notch 1 has been proposed to be related to a functional role of EYS in Notch signalling in muscle regeneration [62] and possibly to other human diseases. Most Notch EGF-repeats have O-glycosylation sites, whose defects have been associated with human diseases [59, 63].
  The effect of EGF O-glycosylation in Notch and other EGF-rich proteins has been mainly studied in Drosophila. Two proteins with multiple EGF O-glycosylation sites involved in retinal disease are Crumbs and Eys [64]. Both proteins are part of a genetic network that determines the apical part of the photoreceptor's development and maintenance in the fly [37]. Loss of O-glycosylation in Eys impairs its extracellular secretion and, consequently, the rhabdomere separation [64]. The enzyme O-glucosyltransferase 1 is essential for Eys' correct folding and its secretion to the extracellular space [64]. Knowing that sugars are only added to properly folded EGF-domains [65], it has been suggested that the O-glycosylation of EGF-domains of Eys might work as quality control and serve to stabilise the folded structure [60].
  The sensitivity of Notch, Crumbs and Eys to defects in EGF O-glycosylation in Drosophila is very different. Notch signalling and Eys function are affected in a similar way, showing a temperature-dependent phenotype indicative of protein misfolding, while Crumbs' trafficking is affected but not its function. Eys and Crumbs biosynthetic traffic share common factors in the fly [66]. Human EYS also harbours multiple O-glycosylation target sites, with 4–5 of them clustered in a similar way to the fly Eys, suggesting that the O-glycosylation regulatory role in this protein might be conserved [64]. In humans, CRB1 and CRB2 contain 13 and 8 O-glycosylation sites, respectively (Fig. 5) [64], and mutations produce RP12 [67] and other retinal dystrophies. Their role is not entirely understood, but CRB proteins are involved in the scaffolding of cell-to-cell adhesion and the structural maintenance of the photoreceptor layer.
  The lack of a murine model for RP25 prevents the complete mechanistic interpretation of the data obtained in Drosophila and zebrafish. The use of patient-derived cellular models and the organoid models of retinal disease are promising tools to advance the understanding of EYS-associated diseases' molecular pathophysiology.
- **Roles of EYS outside the retina**

*EYS* gene, as described in the literature, is mainly expressed in the retina, but it has also been identified in muscle, spinal cord, brain [62], fat, colon, heart [68], and testis [35]. Specific gene products of *EYS*, generated by alternative splicing, maybe playing different roles in different tissues.

A genome-wide association study (GWAS) designed to identify genetic risk factors for severe myopathic conditions caused by statin treatment identified three *EYS* SNPs in intron 26, near the first LamG domain [62]. Statin is a common drug used to control cholesterol blood levels. Although side effects affecting muscle are not rare, a minor fraction of patients develop severe neuromuscular disorders coincident with statin treatment. The authors identified several different *EYS* gene products expressed in the human brain, skeletal muscle and spinal cord in addition to the known retinal transcripts [62]. These results suggested that EYS could intervene in protecting motor neurons and skeletal muscle cells from exercise-induced damage. Isackson and cols. [62] determined that the risk was linked to only one copy of the variant allele, but the effect of the variant in the protein was not studied as no molecular dissection of the EYS isoforms expressed in the affected muscle patients were performed in the study.

It is known that Eys protein in Drosophila plays a role in protecting mechanoreceptor and chemoreceptor organs from hyperosmotic shock by providing stiffness and maintaining cellular integrity and tissue morphogenesis [69]. In humans, it has been proposed that EYS may also protect muscle cells and motor neurons from hyperosmotic damage, which is one of the possible mechanisms of exercise intolerance and statin-induced myopathy [62]. Analysing protein domains to hypothesise a possible mechanistic implication for EYS in muscle, these authors suggested that the N-terminal EGF-domains resemble the structure of Notch1. It has been demonstrated that proteins with these domains bind together, making EYS a candidate to participate in Notch signalling, resulting in developmental defects and several human diseases. Notch signalling is involved in the regeneration of muscle tissue after exercise via satellite-cell differentiation to myoblasts [70]. There are 5 LamG domains with high homology on the C-terminal side of EYS, with the three Agrin LamG domains that function in α-DG binding on the muscle surface [71]. The laminin domains of EYS may link the protein to the extracellular matrix (Fig. 4), making EYS variants influence the regenerative potential of muscle with age.

Other reports have also connected EYS variants to increased risk in non-retinal tissues: A synonymous variant in exon 44 of *EYS* gene, of unpredicted clinical significance, was highlighted in a small study directed to uncover genetic variants associated with Trastuzumab cardiotoxicity [72]. The monoclonal antibody against HER2 is used to treat HER2-positive breast or gastric cancer, but in 5% of patients, the treatment induces cardiotoxicity. This study suggested that EYS may be involved in the signal transduction efficiency of HER2 in cardiac myocytes. However, additional and larger cohorts and functional studies are needed to unravel the mechanistic role of EYS in this susceptibility. Even considering that this data comes from a small study in which the EYS variant is different from the SNP identified in the statin study, it adds information to a possible role of EYS as a protective factor for muscle cells.

Recently, some *EYS* polymorphisms have been reported to be associated with an increased risk for lumbar disc herniation [73], a pathology known to be affected by genetic factors [74]. Considering that an increase in Notch signalling in human degenerated disc tissue has been demonstrated [75], EYS role in lumbar disk maintenance might also be related to Notch signalling.

Being EYS such an ample multi-domain protein, it is not surprising that its different isoforms may play multiple roles in different tissues and developmental stages. With GWAS's advanced analysis capacity, the impact of genetic variants in various health conditions will continue to provide clues about currently unknown EYS functions.

### Future opportunities for the treatment of EYS-retinal dystrophy

**Fig. 6:**
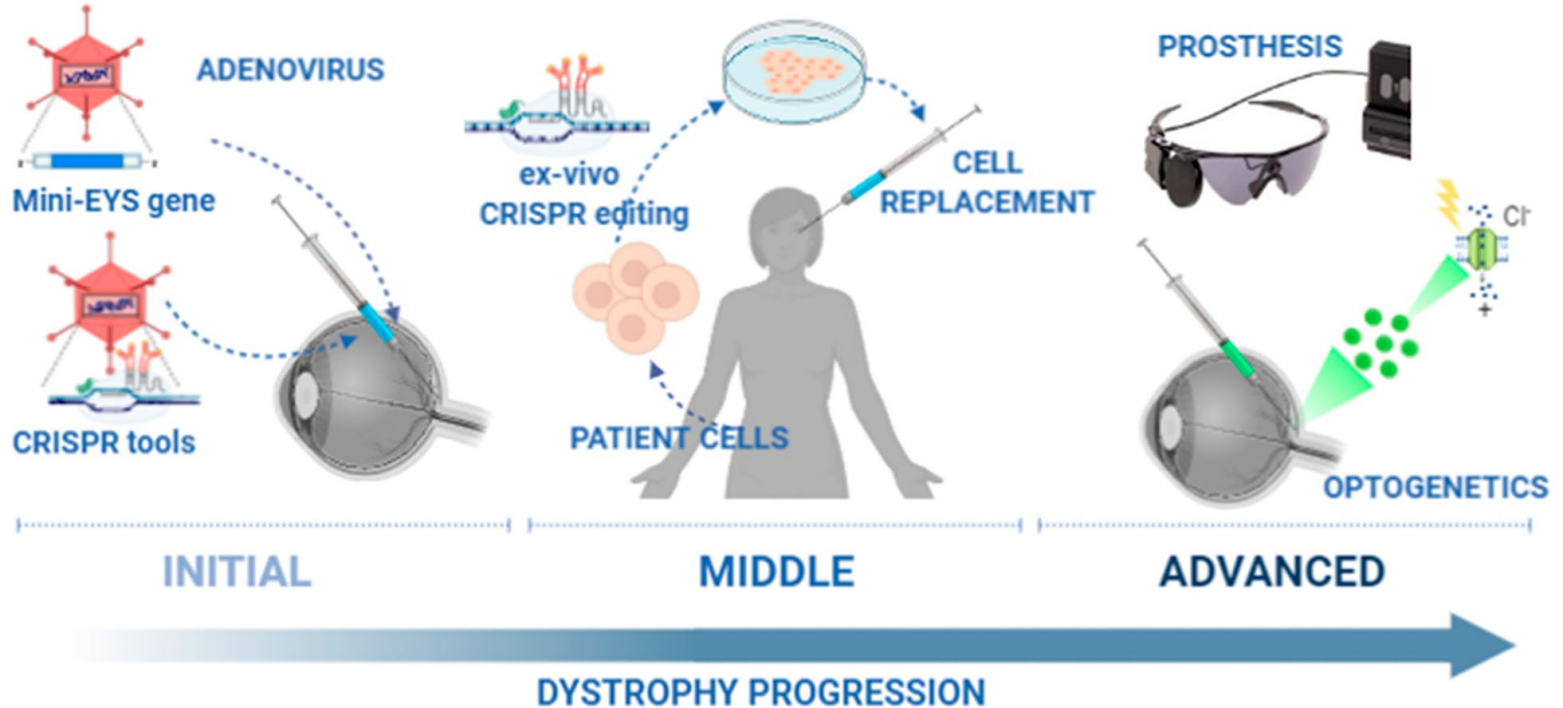
Graphic summary of possible future therapeutic interventions based in advanced therapies for EYS-retinopathy

Although there still is no effective treatment for retinal degenerative diseases, the advances in the knowledge of RP's molecular base have been paralleled by significant progress in the development of novel medical strategies. These strategies can be subdivided into two categories: i) approaches that are gene or mutation-specific and ii) approaches that exert a therapeutic effect independent of the genetic defect [76]. Gene therapy belongs to the first type and can be applied in the early stages of the degenerative process; when the cells in the retina are impaired in their function but the photoreceptor cells can be rescued by external genetic material. In 2008, various research groups reported the first studies testing the safety and efficacy of *RPE65* gene therapy for patients with Leber congenital amaurosis (LCA) caused by a defect in this gene [77–79]. The promising results obtained granted for the recent authorisation in the USA, Europe, Australia and Canada for the first gene-therapy medicinal product. This significant advance in the field supports gene therapy-based research for other genetic retinal dystrophies. Despite these advances, there are still some difficulties in using gene therapy for every genetic defect. One crucial factor is the limited loading capacity of the viral vectors, hindering huge genes' delivery. This is the case for *EYS* and *USH2A* [76]. A strategy to overcome this limitation would be to use a reduced version of the gene to fit in the current valuable vectors, formulating a mini-*EYS* containing only the essential information to prepare a protein able to rescue the healthy phenotype (Fig. 6). Unfortunately, there are still many unknowns about *EYS* and the molecular mechanism of the disease. Further research is needed to design a candidate functional mini-*EYS* for gene therapy. Another possibility for gene therapy would be to use the gene or genes that substitute *EYS* function in the mouse's retina and the other species in which this gene is non-functional or absent once these factors are identified. Additional methods for retinal delivery of transcripts larger than 5 kb using the successful AAVs are theoretically feasible using dual AAV vectors, as reviewed by Trapani [80]. Transgenes of about the size of the *EYS* transcript might be split in two and co-infected in the same cell. To reconstitute the full-length transgene, mechanisms proposed include trans-splicing through concatemerisation of AAV ITRs using donor and acceptor splicing sites or homologous recombination using sequence overlapping. Limitations of these strategies will depend on the efficiency of the specific splicing/DNA recombination events and the frequency of generation of wrong concatemers. To overcome these limitations, a combination of both approaches has been proposed, with hybrid dual AAV vectors.

A recent advance with great potential is the clustered regularly interspaced short palindromic repeats (CRISPR) system, modifying the cell's genome that might be used in situ or ex vivo. This genetic engineering system is being proposed as a future treatment for many disorders originated by a genetic defect. Like *EYS*, gene editing is a promising option for huge genes, given that no exogenous gene has to be supplied to the diseased cells. Instead, their genome will be specifically modified to overcome the defect and produce a functional version of the protein. Although this new technique has been successfully used ex vivo to treat some types of haematological cancers, research in retinal diseases is currently at the preclinical level [81, 82]. There are many open questions yet to be solved before this new approach can be considered safe for non-life-threatening diseases. Not only technical aspects should be considered, such as specificity, efficacy, delivery or immune response to the nucleases, but also the obvious concerns about the risks of a nuclease that is delivered into the cell to break and modify the genome.

Regarding the non-specific, mutation-independent approaches, a treatment proposed for degenerative diseases is cell replacement. This, indeed, might be the appropriate option for advanced retinal dystrophy, with extensive damage in the cells of the retina, which will not benefit from a gene-rescue. Nowadays, a significant amount of research is devoted to the development of retinal cells manufactured for regenerative medicine. The biological material source can be either ocular-derived retinal progenitor cells or cells differentiated in vitro from embryonic stem cells or induced pluripotent stem cells (iPSCs). For patient-derived iPSCs, CRISPR genome editing strategies should be integrated into the therapeutic approach to correct genetic defects before transplantation [76, 83, 84]. Although this is a complicated approach, it would provide the added benefit of full immunological compatibility between the host and the implant (Fig. 6). It is worth considering that, for diseases in which the primary cell type affected is RPE, cell replacement therapy is closer to being available. Years of preclinical studies have provided a firm base in which some small clinical trials have shown safety [85, 86]. On the other side, for diseases that require the replacement of photoreceptors, as is the case of EYS-retinopathy, there are still several technical issues to solve. Cell culture protocols to obtain photoreceptor precursors are much more complicated than RPE differentiation protocols, generating a mixture of cell types from which the photoreceptor progenitors need to be selected. Moreover, a successful graft does require not only the survival of the cells but also the neural connection of the exogenous cellular material to the host retinal neuronal network. This is a challenge that many research teams worldwide are now pursuing. Although new advances are constantly shown, photoreceptor cell therapy still needs time to become a reality.

For very advanced RP cases, prosthetics is another option (Fig. 6). An electronic retinal implant can provide artificial vision. The Argus II epiretinal implant (Second Sight Medical Products) has been approved by the US FDA and the European CE certification, and Alpha IMS subretinal implant (Retina Implant AG) has been approved by European CE certification [87]. Artificial vision does not produce the same type of visual information as the natural retinal function but can help some blind people to gain navigation autonomy after rehabilitation and learning. A more sophisticated prosthetics type is optogenetics, in which surviving retinal cells that are non-photosensitive are transformed to be light-sensitive via genetic modification. Promising results have been shown in cell-based models and animal models, but it has still to be proven in clinical trials [88]. In Europe, the PIONEER phase I/IIa clinical trial (NCT03326336) is currently ongoing, combining optogenetics directed to remaining ganglion cells in the retina with a light-amplification device.

Finally, trying to intervene in the neurodegenerative process induced by RP is another possibility. Neuroprotection can extend the life of the photoreceptors, delaying the course of the disease. It also might increase the possibilities of the success of gene or cell therapy. Several candidates have been tested as neuroprotective factors, including basic fibroblast growth factor (bFGF), brain-derived neurotrophic factor (BNDF), ciliary neurotrophic factor (CNTF), glial cell-derived neurotrophic factor (GNDF) and rod-derived cone viability factor (rdCVF), and all of them have shown increased photoreceptor survival in animal models and humans [89–91].

## Conclusions

Intrinsic difficulties associated with the study of EYS-retinopathy include the size of the gene and the absence of a mouse or rat animal model of the disease. Combining the data obtained from other animal models, EYS has been localised to the interphotoreceptor matrix, and its defects have been associated with ciliary dysfunction. EYS is the first extracellular protein known to cause ciliopathy, and other possible roles and interactions are still under study. Further knowledge of the mechanism of action at the molecular and cellular level will help design specific advanced therapies for EYS-retinopathy.

## Data Availability

Not applicable.

## Abbreviations

arCRD: Autosomal recessive cone-rod dystrophy
arRP: Autosomal recessive retinitis pigmentosa
bFGF: Basic fibroblast growth factor
BNDF: Brain-derived neurotrophic factor
CNTF: Ciliary neurotrophic factor
CNV: Copy-number variation
CRISPR: Clustered regularly interspaced short palindromic repeats
DG: Dystroglycan
EGF: Epithelial growth factor
ERG: Electroretinogram
EZ: Ellipsoid zone
FAF: Fundus autofluorescence
GNDF: Glial cell-derived neurotrophic factor
GWAS: Genome-wide association study
IPM: Interphotoreceptor matrix
iPSC: Induced pluripotent stem cells
IRD: Inherited retinal degeneration
Lam: Laminin
LCA: Leber congenital amaurosis
NMD: Nonsense-mediated decay
OCT: Optical coherence tomography
OS: Outer segments
rdCVF: Rod-derived cone viability factor
RP: Retinitis pigmentosa
RPE: Retinal pigment epithelium
SNPs: Single nucleotide polymorphisms
